# Position-Constrained Calibration Compensation for Hand–Eye Calibration in Industrial Robots

**DOI:** 10.3390/s24237554

**Published:** 2024-11-26

**Authors:** Jinsong Lin, Yuxing Feng, Wenze Ren, Jiahui Feng, Jun Zheng

**Affiliations:** Department of Mechanical Engineering, Tsinghua University, Beijing 100084, China; linjs22@mails.tsinghua.edu.cn (J.L.); zndxfyx@163.com (Y.F.); fjh_1@mail.tsinghua.edu.cn (J.F.)

**Keywords:** line-structured light sensor, hand-eye calibration, welding robots, spherical constraints

## Abstract

The hand–eye calibration of laser profilers and industrial robots is a critical component of the laser vision system in welding applications. To improve calibration accuracy and efficiency, this study proposes a position-constrained calibration compensation algorithm aimed at optimizing the hand–eye transformation matrix. Initially, the laser profiler is mounted on the robot and used to scan a standard sphere from various poses to obtain the theoretical center coordinates of the sphere, which are then utilized to compute the hand–eye transformation matrix. Subsequently, the positional data of the standard sphere’s surface are collected at different poses using the welding gun tip mounted on the robot, allowing for the fitting of the sphere’s center coordinates as calibration values. Finally, by minimizing the error between the theoretical and calibrated sphere center coordinates, the optimal hand–eye transformation matrix is derived. Experimental results demonstrate that, following error compensation, the average distance error in hand–eye calibration decreased from 4.5731 mm to 0.7069 mm, indicating that the proposed calibration method is both reliable and effective.

## 1. Introduction

Robotic welding technology is essential in modern manufacturing, particularly in the automotive, shipbuilding, and pressure vessel industries, where automated welding is in high demand [1,2,3]. The line-structured light sensor, valued for its non-contact measurement, high speed, accuracy, and stability, has emerged as the preferred tool for automated robotic welding [4,5]. A prevalent measurement technique involves affixing a line-structured laser sensor to the end effector of a robot. This method capitalizes on the robot’s inherent flexibility, allowing for a wider range of measurements than a sensor that is stationary [6].

Upon the initial installation of the sensor on the robot flange, it is essential to ascertain the relative pose relationship between the sensor and the robot’s end effector, a process referred to as hand–eye calibration [7]. The accuracy of hand–eye calibration directly impacts the precision of the sensor’s measurements in the robot’s coordinate system, which is crucial for high-precision tasks such as weld seam detection and tracking. The standard approach of hand–eye calibration is to formulate a matrix equation derived from the robot’s kinematic model, employing calibration objects as constraints to determine the transformation matrix that relates the sensor coordinate system to the end effector [8,9,10,11,12,13]. However, during hand–eye calibration, error accumulation occurs due to sensor calibration errors, geometric errors and motion errors of the robotic arm, and deformation errors caused by load gravity. Therefore, the calibration scheme requires urgent optimization.

The accuracy of hand–eye calibration directly impacts the precision of the entire robotic vision system. This challenge has led many experts and scholars to explore algorithms and models to effectively improve the accuracy of robot hand–eye calibration. Shiu and Ahmad [14] initially converted hand–eye calibration into the problem of solving the rotation matrix, as well as solving the nonlinear homogeneous equation **AX = BX**. However, since the line-structured light sensor outputs two-dimensional point cloud data, Huang et al. [15] and Yang et al. [16] used the center of a stationary standard sphere as a constraint to establish and solve the matrix equation of the form **AX = B** instead of **AX = XB**. An et al. [17] proposed a combined optimization method to address the impact of random errors during hand–eye calibration and applied it to the hand–eye calibration process, However, this method did not consider the motion and geometric errors of the robotic arm, nor did it include the installation of an end effector. Cao et al. [18] proposed a simultaneous calibration method for the robot hand–eye relationship and kinematics using a line-structured light sensor. They utilized two standard spheres with known center distances for calibration, reducing the average distance error from 3.967 mm to 1.001 mm. Still, this method did not account for non-kinematic errors caused by load, and the calibration model and process were complex and inefficient. Furthermore, existing hand–eye calibration algorithms do not account for cumulative errors, rendering them inadequate for weld seam detection requirements.

To address these challenges, this paper proposes a compensation algorithm based on position constraints to optimize the calibration model parameters. An experimental platform is constructed to verify the correctness and effectiveness of the proposed methods. This paper is organized as follows: Section 2 introduces the principles of hand–eye calibration and error compensation techniques. Section 3 describes the industrial robot measurement system and presents the experimental results, including a comprehensive calibration process, error compensation procedures, and verification of the optimized hand–eye transformation matrix. Finally, Section 4 provides a summary of the paper and presents the concluding remarks.

## 2. Hand–Eye Calibration of the Line Structure Light Sensor

Using the 3D measurement data from the line-structured light sensor, the trajectory of the robotic arm equipped with a welding torch can be planned to achieve automated welding. The key to this technology is accurately calibrating the rigid body transformation from the coordinate system $O_{C}-X_{C}Y_{C}Z_{C}$ to the robotic arm tool coordinate system $O_{TO}-X_{TO}Y_{TO}Z_{TO}$, i.e., calibrating the hand–eye matrices for the sensor used in industrial robots.

### 2.1. Principle of the Hand–Eye Calibration Algorithm Based on Spherical Constraints

**Figure 1:**
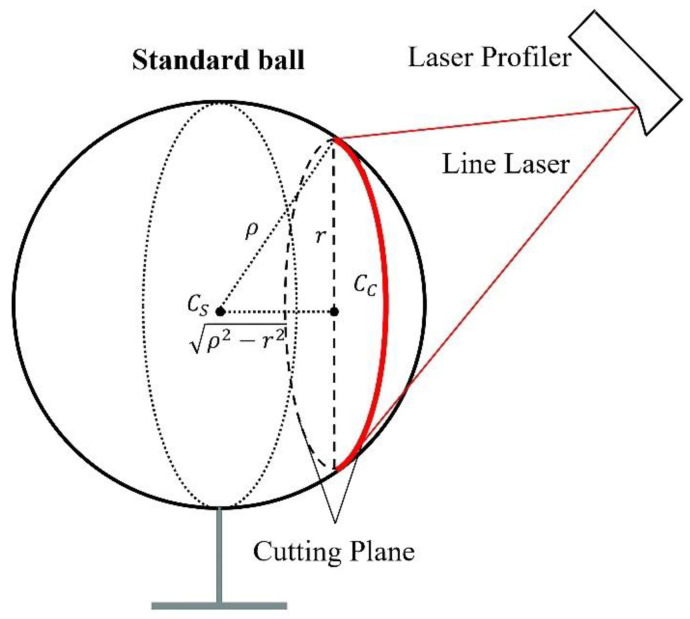
Calibration sphere model.

For an automated welding system, hand–eye calibration is the process of calibrating the relative coordinate transformation between the welding torch tip and the camera. A standard sphere fixed in the robot’s workspace is used as the calibration target. When the laser line emitted by the sensor projects onto the surface of the standard sphere, the intersection forms a circle, and the laser line creates a circular arc, as shown in Figure 1.

Since the circle’s center $C_{c}$ lies in the light plane, its coordinates in the $O_{C}-X_{C}Y_{C}Z_{C}$ system can be represented as $\left(x_{cc},0,z_{cc}\right)$. By extracting the arc points and fitting the circle, the values of $x_{cc}$, $z_{cc}$, and the radius *r* can be obtained. Using the Pythagorean theorem and considering the geometric properties of the sphere, the coordinates of the sphere’s center $C_{s}$ in the $O_{C}-X_{C}Y_{C}Z_{C}$ system can be expressed as $\left(x_{cs},y_{cs},z_{cs}\right)$ as follows [17]:

(1) $$\left\{\begin{array}{c} x_{cs}=x_{cc}\\ y_{cs}=\pm \sqrt{\rho^{2}-r^{2}}\\ z_{cs}=z_{cc} \end{array}\right.$$

Here, ρ is the radius of the sphere, and the sign of $y_{cs}$ is determined by the relative position of the line-structured light sensor and the standard sphere.

By keeping the sphere’s center fixed and changing the sensor’s position and orientation multiple times, the coordinates of the sphere center in the $O_{C}-X_{C}Y_{C}Z_{C}\;$ system are obtained as $P_{csk}\left(x_{csk},y_{csk},z_{csk}\right)$, where *k* denotes the *k*-th measurement state of the robotic arm at different positions and orientations. According to the rigid body transformation, the relationship between the sphere center’s position in the robotic arm base coordinate system $O_{B}-X_{B}Y_{B}Z_{B}$ and $P_{csk}$ is given as follows:

(2) $$\left[\begin{array}{c} P_{fb}\\ 1 \end{array}\right]=\left[\begin{array}{cc} R_{bk} & T_{bk}\\ 0 & 1 \end{array}\right]\left[\begin{array}{cc} R_{to} & T_{to}\\ 0 & 1 \end{array}\right]\left[\begin{array}{c} P_{csk}\\ 1 \end{array}\right]$$

Here, $R_{bk}$ (3 × 3) and $T_{bk}$ (3 × 1) are determined by the robotic arm’s calibration algorithm and are considered known. $R_{to}\left(3\times 3\right)$ and $T_{to}$ (3 × 1) are the hand–eye matrices to be solved.

First, solve for the rotation matrix $R_{to}$, ince the robotic arm only undergoes translational motion and does not change its orientation between two positions, the rotation matrix $R_{bk}$ remains constant [17], i.e., $R_{b1}=R_{b2}$. Based on this, the following equation can be derived:

(3) $$R_{to}\left(P_{cs1}-P_{cs2}\right)=R_{b1}^{-1}\left(T_{b2}-T_{b1}\right)=R_{b1}^{T}\left(T_{b2}-T_{b1}\right)$$

Here, $R_{b1}^{-1}$ and $R_{b1}^{T}$ are the inverse and transpose of $R_{b1}$, respectively. Since $R_{b1}$ is an orthogonal matrix, $R_{b1}^{-1}=R_{b1}^{T}$.

After moving the robotic arm through $n_{t}\left(n_{t}\geq 4\right)$ translations and measuring $P_{csk}$, $n_{t}$ − 1 equations can be established from Equation (3):

$$R_{to}A=B,$$

$$A=\left[\begin{array}{c} P_{cs1}-P_{cs2}\\ P_{cs1}-P_{cs3}\\ \ldots \\ P_{cs1}-P_{csn_{t}} \end{array}\right],$$

(4) $$B=\left[\begin{array}{c} R_{b1}^{T}\left(T_{b2}-T_{b1}\right)\\ R_{b1}^{T}\left(T_{b3}-T_{b1}\right)\\ \ldots \\ R_{b1}^{T}\left(T_{{bn}_{t}}-T_{b1}\right) \end{array}\right]\cdot$$

To obtain the optimal solution for the rotation matrix $R_{to}$, following the idea of the Iterative Closest Point (ICP) registration algorithm [19], let $H_{ICP}=AB^{T}$. Performing SVD [20] on $H_{ICP}$ yields $H_{ICP}=U_{ICP}\Sigma_{ICP}V_{ICP}^{T}$. The optimal rotation matrix based on the measurement results is then the following:

(5) $$R_{to}=V_{ICP}U_{ICP}^{T}$$

The translation vector $T_{to}$ can be obtained by changing the position and orientation of the robotic arm through arbitrary rotations and translations. From Equation (2), the following can be derived:

(6) $$\left(R_{b1}-R_{b2}\right)T_{to}=R_{b2}R_{to}P_{cs2}-R_{b1}R_{to}P_{cs1}+T_{b2}-T_{b1}$$

By changing the robotic arm’s position and orientation $n_{r}\left(n_{r}\geq 4\right)$ times and measuring $P_{csk}$, the following can be obtained:

$$CT_{to}=D,$$

$$C\;=\;\left[\begin{array}{c} R_{b1}-R_{b2}\\ R_{b1}-R_{b3}\\ \ldots \\ R_{b1}-R_{{bn}_{r}} \end{array}\right],$$

(7) $$D\;=\;\left[\begin{array}{c} R_{b2}R_{to}P_{cs2}-R_{b1}R_{to}P_{cs1}+T_{b2}-T_{b1}\\ R_{b3}R_{to}P_{cs3}-R_{b1}R_{to}P_{cs1}+T_{b3}-T_{b1}\\ \ldots \\ R_{{bn}_{r}}R_{to}P_{{csn}_{r}}-R_{b1}R_{to}P_{cs1}+T_{{bn}_{r}}-T_{b1} \end{array}\right]\cdot$$

Since the coefficient matrix C is known, the optimal translation vector can be obtained using the least squares method:

(8) $$T_{to}={\left(C^{T}C\right)}^{-1}C^{T}D$$

### 2.2. Position-Constrained Calibration Compensation Algorithm

The aforementioned hand–eye calibration algorithm inevitably encounters errors due to several factors: (a) When solving for the hand–eye parameters, the calibration equations are established based on motion constraints, assuming that the sensor detection data are unbiased. However, errors exist in the sensor’s intrinsic calibration. (b) The robotic arm itself has geometric and motion errors, and relying solely on theoretical matrix decomposition has limitations. (c) The robotic arm, equipped with a welding torch and the sensor, undergoes deformation due to gravity, resulting in deformation errors. These errors contribute to inaccuracies in the theoretical matrix decomposition process, making it challenging to ensure the precision of experimental calibration. To address this issue, a hand–eye calibration error compensation algorithm is proposed:

Step 1: Use the hand–eye parameters $R_{to}$ and $T_{to}$ obtained from the hand–eye calibration algorithm described in Section 2.1 as the initial solution.

Step 2: Control the robotic arm, equipped with the sensor and welding torch, to collect positional information on the standard sphere’s surface points from various orientations at multiple poses. Fit the coordinates of $C_{s}$ in the $O_{B}-X_{B}Y_{B}Z_{B}$ system to obtain $P_{fb}$, which represents the true position of $C_{s}$ in the $O_{B}-X_{B}Y_{B}Z_{B}$ system.

Step 3: Substitute the sphere center position $P_{csk}$ measured at the *k*-th pose and the pose matrices $R_{bk}$ and $T_{bk}$ relative to the $O_{B}-X_{B}Y_{B}Z_{B}$ system into Equation (2) to obtain the theoretical position ${\hat{P}}_{fb}$ of $C_{s}$ in the $O_{B}-X_{B}Y_{B}Z_{B}$ system.

Step 4: Based on the principle of minimizing the error between the theoretical sphere center coordinates and the true sphere center coordinates, establish the objective function of Equation (9). Use the LM algorithm to iteratively solve Equation (9), optimizing the hand–eye parameters $R_{to}$ and $T_{to}$ of the robot vision-guided system until the results converge.

(9) $$min\sum_{k=1}^{n_{com}}\|P_{fb}-{\hat{P}}_{fb}{\|}^{2}$$

where $n_{com}$ is the total number of times the sensor measures the sphere center when obtaining the initial hand–eye parameters, which is the sum of the number of translations $n_{t}$ and the number of arbitrary pose changes $n_{r}$ of the robotic arm.

## 3. Experimental Verification and Results Analysis

### 3.1. Experimental Setup

**Figure 2:**
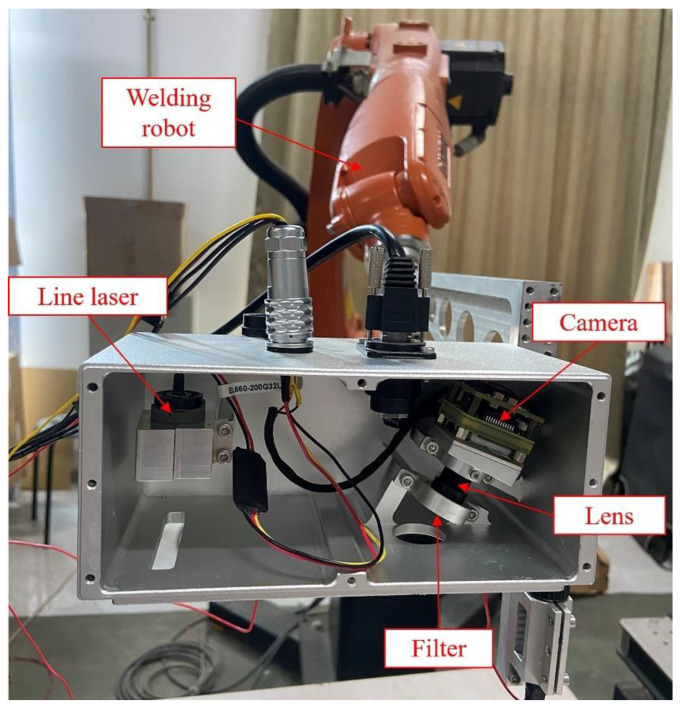
Line-Structured light sensor and robot system.

The line-structured light sensor and the robotic arm used in this system is shown in Figure 2. The camera is a board-level CMOS camera, model MV-CB016-10GM-C from Hikvision (Hangzhou, China), with a resolution of 1440 × 1080 and a pixel size of 3.45 μm × 3.45 μm. It is equipped with a fixed-focus lens with a focal length of 12 mm. Considering the ambient light conditions in the application environment, a laser emitter with a wavelength of 658 ± 5 nm was selected, and a narrow-band filter matching this wavelength range was installed in front of the lens. The robotic arm used is a KUKA KR5 R1400 (Augsburg, Bavaria, Germany), a six-axis industrial robot.

### 3.2. Hand–Eye Calibration Experiment

In the hand–eye calibration experiment, a calibration tool—hereinafter referred to as the “simulated welding torch”—was employed to replicate the operation of an actual welding torch. A standard stainless-steel ball, with a diameter of 40.00 mm ± 0.01 mm, was securely positioned within the workspace of the robotic arm’s vision guidance system. According to the calibration procedure detailed in Section 2.1, the robotic arm was first controlled to measure the standard ball 10 times using the line-structured light sensor, restricting movement to translation only. Subsequently, the robotic arm measured the standard ball another 10 times using the line-structured light sensor from arbitrary poses [17]. During these measurements, to prevent singularities in the calibration algorithm, variations occur along the $X_{B}$, $Y_{B}$, and $Z_{B}$ axes of the base coordinate system were ensured when translating the robotic arm. When the robotic arm adopted arbitrary poses, all six joints exhibited variation. Finally, the hand–eye matrices $R_{to}$ and $T_{to}$ were determined:

(10) $$\left\{\begin{array}{c} R_{to}=\left[\begin{array}{ccc} -0.0383 & -0.0495 & 0.9980\\ -0.0641 & 0.9968 & 0.0470\\ -0.9972 & -0.0622 & -0.0414 \end{array}\right],\\ T_{to}=\left[\begin{array}{c} -162.085\\ -173.945\\ 60.7321 \end{array}\right]\cdot \end{array}\right.$$

After completing the above steps, the robotic arm’s pose was adjusted to measure the positions of three points using the line-structured light sensor from ten different viewpoints. The deviation between the sensor’s measured positions and the actual positions was calculated. The average deviations were 4.3132 mm for point 1, 4.5731 mm for point 2, and 4.0331 mm for point 3, as shown in Table 1. These values are significantly higher than the requirement for industrial robot vision guidance systems for weld tracking, which is within 1.5 mm, indicating the need for further optimization.

**Figure 3:**
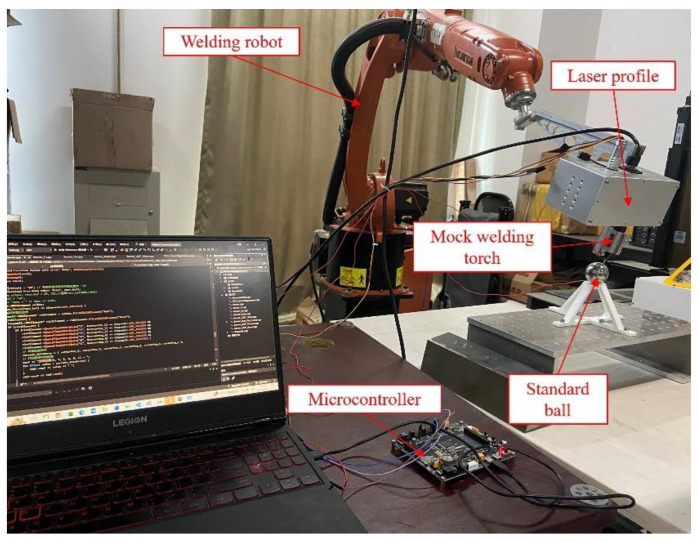
Calibration error compensation system.

To address the insufficient accuracy, 60 sets of standard sphere surface points were randomly collected within the robot’s workspace. A microcontroller connected the robotic arm and the standard sphere to ensure accuracy. The robotic arm touching the standard sphere served as a trigger signal to collect the real-time position of the mock welding torch tip at the end of the robotic arm, as shown in Figure 3. The collected sphere points were used to fit the sphere expression, thereby identifying the accurate position of the sphere center.

Following the hand–eye calibration compensation algorithm based on position constraints described in Section 2.2, the optimized hand–eye parameters $R_{t}$ and $T_{t}$ were obtained. The positions of the three points were subsequently remeasured using a line-structured light sensor from ten consistent viewpoints during the calibration process. Table 1 presents the average errors of the feature points before and after error compensation. The results revealed that the average deviation was 0.8183 mm for point 1, 0.7069 mm for point 2, and 0.6371 mm for point 3. These deviations are well below 1.5 mm, meeting the requirements for weld seam tracking.

**Figure 4:**
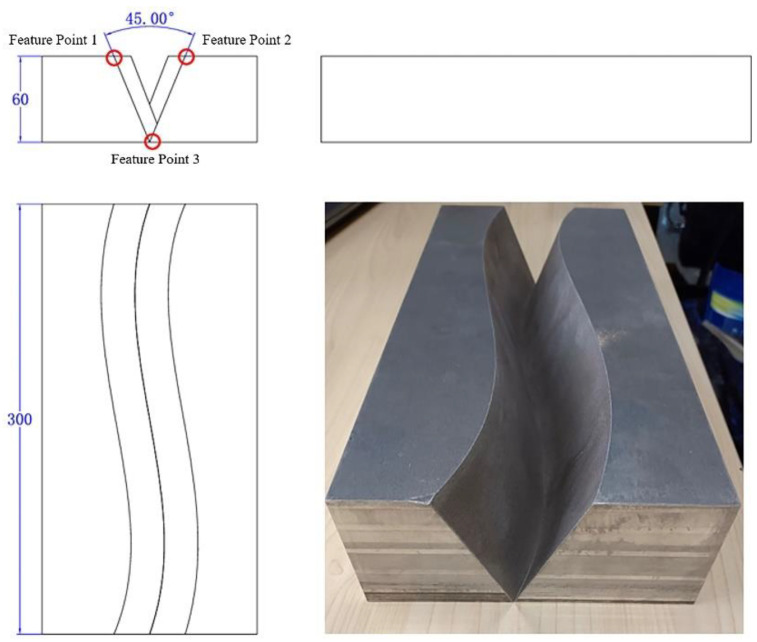
V-groove weld of S-shaped trajectory.

To verify the accuracy of the proposed algorithm for detecting weld seams and guide welding in the thick plate welding, an S-shaped trajectory V-groove welding piece was designed and manufactured, as shown in Figure 4. This V-groove welding piece had a thickness of 60 mm, a length of 300 mm, and a groove angle of 45°.

**Figure 5:**
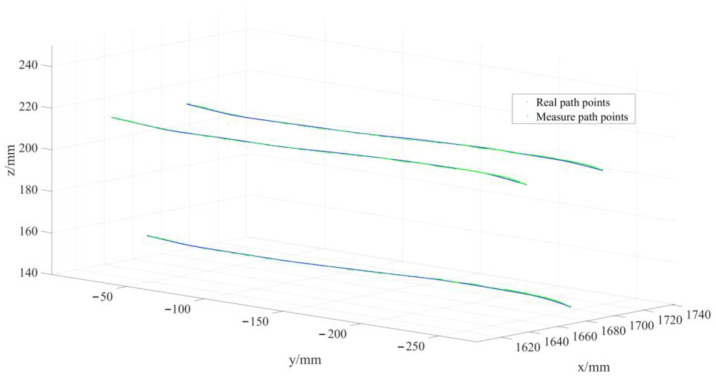
Comparison of weld detection trajectory and actual trajectory.

**Figure 6:**
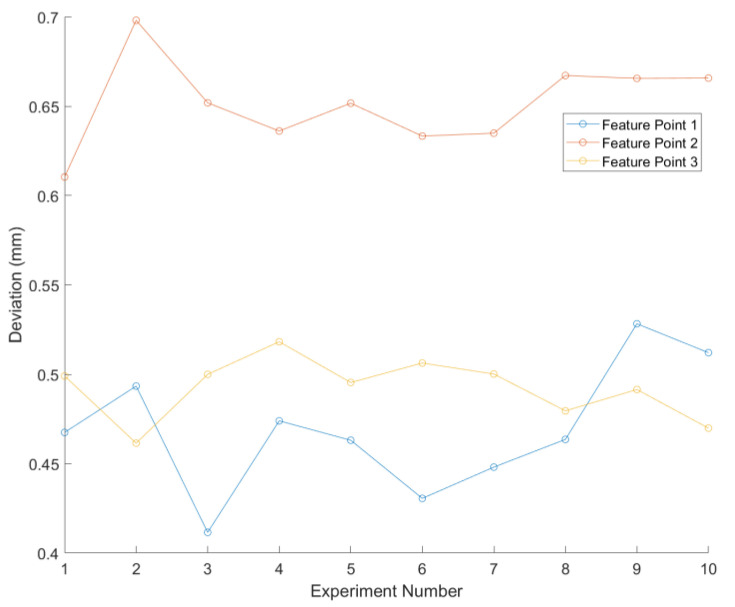
Average deviation results of feature points.

By comparing all V-groove weld seam feature points detected by the sensor during tracking with the actual weld seam trajectory, as illustrated in Figure 5, the average absolute trajectory error was determined. Figure 6 presents the deviations between the measured and actual values of the trajectories for the three feature points across ten measurement sessions. The average errors for the weld seam trajectory were 0.4567 mm for feature point 1, 0.6374 mm for feature point 2, and 0.4856 mm for feature point 3, as detailed in Table 2. These errors meet the application requirements for effective weld seam tracking.

### 3.3. Three-Dimensional Reconstruction Accuracy Verification

**Figure 7:**
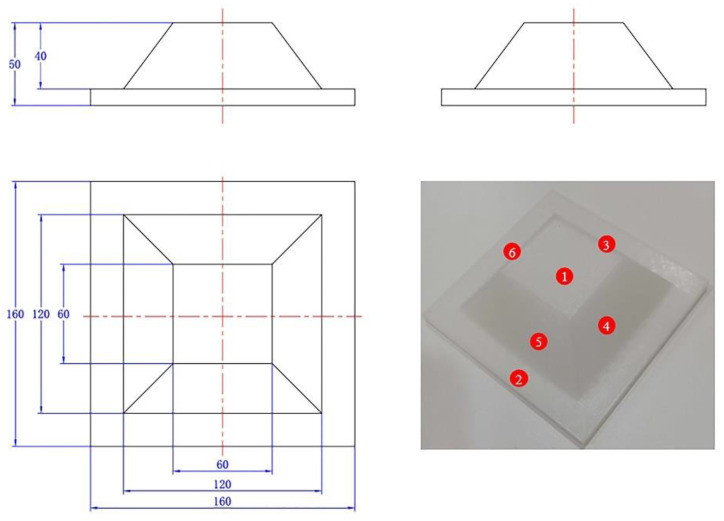
Boss for evaluating 3D reconstruction accuracy. The numbers 1 to 6 correspond to the six planes of the convex platform, respectively.

**Figure 8:**
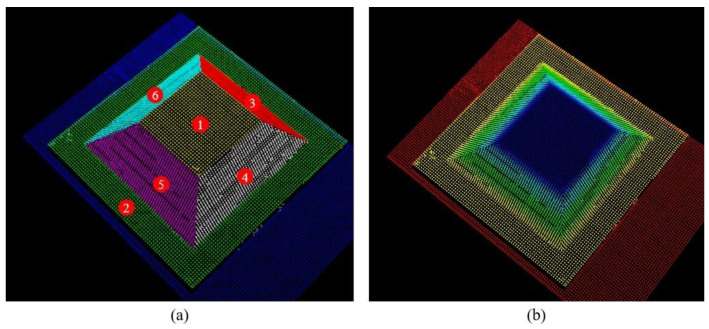
3D reconstruction results of boss: (**a**) boss point cloud; (**b**) point cloud segmentation. The numbers 1 to 6 correspond to the six planes of the convex platform, respectively.

To further validate the generalizability of the algorithm, a regular boss with a dimensional accuracy of 0.01 mm was designed as the evaluation object. The calibrated robot and the laser vision sensor were then employed to perform the 3D reconstruction. Different numerical labels were assigned to each plane of the boss, as depicted in Figure 7. The results of scanning and reconstructing the boss using the line-structured light sensor are shown in Figure 8a. The point cloud quality is high and meets the evaluation requirements. Additionally, the point cloud of the boss was segmented into planes, each assigned different colors to distinguish the planes corresponding to the numerical labels, as shown in Figure 8b.

To quantitatively evaluate the reconstruction accuracy, both length and angle measurements were considered. The four slanted edges of the boss were used as the length evaluation standard. According to the design dimensions in Figure 7, the theoretical lengths of the slanted edges $L_{34}$, $L_{45}$, $L_{56}$, and $L_{63}$ are 50 mm. The dihedral angles between plane 2 and the four slanted surfaces were used as the angle evaluation standard, with theoretical values of $\theta_{23}$, $\theta_{24}$, $\theta_{25}$, and $\theta_{26}$ being 53.13°. After segmenting the reconstructed point cloud of the boss into planes and performing geometric calculations, the deviations between the point cloud data and the theoretical data were obtained, as shown in Table 3. The average length deviation was 0.5071 mm, and the average angle deviation is 0.2145°, both of which meet the application requirements for weld seam tracking.

## 4. Conclusions

A comprehensive and effective calibration scheme was proposed for a line-structured light sensor based on a constant-focus optical path. By introducing a two-dimensional tilt angle, a more suitable inclined camera imaging model for the line-structured light sensor was established. The detailed process of obtaining initial values and performing nonlinear optimization of the model parameters was thoroughly described. Using a dual-step target as a marker, numerous non-collinear points on the light plane were quickly and conveniently obtained, and the light plane equation in the ideal camera coordinate system was accurately fitted. An integrated automatic calibration device was designed to efficiently complete the calibration experiments of the vision sensor while adhering to imaging constraints.

The experimental results demonstrate that the average distance error of the hand–eye calibration after error compensation decreased from 4.5731 mm to 0.7069 mm. This indicates that the proposed calibration method ensures high detection accuracy and repeatability for the line-structured light sensor. Additionally, the effectiveness of the calibration method in reducing the impact of hand–eye calibration errors for industrial robots was validated.

## Figures and Tables

**Table 1:** Average deviation of feature points.

Feature Points	Feature Points 1	Feature Points 2	Feature Points 3
Average Deviation (mm) After Calibration	4.3132	4.5731	4.0331
Average Deviation (mm) After Compensation	0.8183	0.7069	0.6371

**Table 2:** Average deviation of weld seam feature points.

Feature Points	Feature Point 1	Feature Point 2	Feature Point 3
Average Deviation (mm)	0.4567	0.6374	0.4856

**Table 3:** Deviations between point cloud data and theoretical data.

Length	Point Cloud (mm)	Deviation (mm)	Angle Parameters	Point Cloud Data (°)
$L_{34}$	50.3947	0.3947	$\theta_{23}$	53.4567
$L_{45}$	50.4891	0.5109	$\theta_{24}$	53.0064
$L_{56}$	50.6713	0.6713	$\theta_{25}$	53.3356
$L_{63}$	50.4513	0.4513	$\theta_{26}$	52.9278
Average	50.2516	0.5071	Average	53.1816

## Data Availability

The data presented in this study are available on request from the corresponding author.
